# Integration of Palliative Care Into Comprehensive Cancer Treatment at Moi Teaching and Referral Hospital in Western Kenya

**DOI:** 10.1200/JGO.2015.000125

**Published:** 2015-09-23

**Authors:** Kenneth Cornetta, Susan Kipsang, Gregory Gramelspacher, Eunyoung Choi, Colleen Brown, Adam B. Hill, Patrick J. Loehrer, Naftali Busakhala, F. Chite Asirwa

**Affiliations:** **Kenneth Cornetta, Gregory Gramelspacher, Eunyoung Choi, Adam B. Hill, Patrick J. Loehrer**, and **F. Chite Asirwa**, School of Medicine, Indiana University; **Colleen Brown**, Palliative Care Program, St. Vincent Indianapolis Hospital, Indianapolis, IN; **Susan Kipsang, Naftali Busakhala**, and **F. Chite Asirwa**, Moi Teaching and Referral Hospital; and **Naftali Busakhala** and **F. Chite Asirwa**, Moi University, Eldoret, Kenya.

## Abstract

**Purpose:**

The prognosis for the majority of patients with cancer in Kenya is poor, with most patients presenting with advanced disease. In addition, many patients are unable to afford the optimal therapies required. Therefore, palliative care is an essential part of comprehensive cancer care. This study reviews the implementation of a palliative care service based at the Moi Teaching and Referral Hospital in Eldoret, Kenya, and describes the current scope and challenges of providing palliative care services in an East African tertiary public referral hospital.

**Methods:**

This is a review of the palliative care clinical services at the only tertiary public referral hospital in western Kenya from January 2012 through September 2014. Palliative care team members documented each patient's encounter on standardized palliative care assessment forms; data were then entered into the Academic Model Providing Access to Health Care (AMPATH)-Oncology database. Interviews were also conducted to identify current challenges and opportunities for program improvement.

**Results:**

This study documents the implementation of a palliative care service line in Eldoret, Kenya. Barriers to providing optimal palliative cancer care include distance to pharmacies that stock opioids, limited selection of opioid preparations, education of health care workers in palliative care, access to palliative chemoradiation, and limited availability of outpatient and inpatient hospice services.

**Conclusion:**

Palliative care services in Eldoret, Kenya, have become a key component of its comprehensive cancer treatment program.

## INTRODUCTION

The prognosis for patients with cancer in low- and middle-income countries (LMICs) is markedly different from that for individuals living in high-income countries (HICs). In 2008, the estimated death rate from cancer in the United States was 40% compared with 79% in Eastern Africa.^1^ A variety of organizations, including the Kenyan Ministry of Health, have identified the need for investment in comprehensive cancer care.^2–9^ To date, implementation of cancer care has been undertaken in collaborative efforts between LMIC health ministries and HIC governmental and nongovernmental agencies. One such effort is Academic Model Providing Access to Health Care (AMPATH)-Oncology. This program followed the lead of its parent organization AMPATH, which developed a successful platform to address HIV in western Kenya.^10,11^ Both AMPATH and AMPATH-Oncology represent a collaboration between Moi University School of Medicine, Moi Teaching and Referral Hospital (MTRH), and a consortium of North American academic medical centers.

The initial aim of AMPATH-Oncology was to treat children with curable cancers and HIV-positive adults with malignancies. The program began seeing patients with non-HIV–related cancers in 2008 and had evaluated more than 30,000 patients for cancer screening and treatment by 2012. Addressing the needs of patients with cancer highlighted the need for palliative care services; here, we describe the implementation of a palliative care program for adult and pediatric patients with cancer.

## METHODS

A palliative care clinical service was implemented within a public hospital in Eldoret, Kenya. Palliative care team members documented new and return patient visits by using a standardized four-page palliative care assessment form. The components of the assessment form are provided in Table 1. Data forms are manually entered into the AMPATH-Oncology database, and summary data are presented in this article. AMPATH-Oncology approved release of the deidentified data set for services delivered from January 2012 through September 2014. A qualitative assessment of the program was performed by asking members of the MTRH palliative care team to identify the major strengths and challenges of the existing clinical services.

**Table 1:** New Patient Intake Form

Demographics
Name, date of birth, sex, tribe
Insurance status
Contact information
Religion
Marital status
Speaking language
Education attainment
Occupation of patient
Occupation of spouse/parent/guardian
Main source of income/financial support
Oncology clinic site
Medical history
Major diagnosis
Primary site/metastatic sites
HIV status
Past medical conditions
Surgeries
Chemotherapy/radiotherapy
Hormone therapy
Herbal therapy
Reason for referral
Anticipated prognosis
Patient and family understanding of the illness
Medications
Allergies and adverse reactions
Present medications
Discontinued medications
Alcohol/tobacco/recreational drugs
Psychosocial assessment
Genogram of immediate family
Identification of major physical caregiver
Identification of major financial caregiver
Spiritual
Hope/peace/origin of power
Divine/religious/spiritual rituals
Relationship between spirituality and illness
Staff assessment and plan
Main distress for patient
Main distress for family
Patient's goals and expectations, including physical, psychosocial, and spiritual
Caregiver's goals and expectations, including physical, psychosocial, and spiritual

## RESULTS

### Program Development and Growth

For patients with cancer at MTRH, challenges to care include access to opioids, communication of prognostic information, and financial and faith-based barriers to patients' acceptance of palliative care. After securing financial support from MTRH, AMPATH-Oncology, and other philanthropic organizations, the palliative care service began seeing patients in 2010. The initial program included nurses and social workers. A full-time clinical officer was added to the team in 2012 (clinical officers fulfill the role of physician assistants) and a full-time medical officer was added in 2014. The composition of the current team is described in Table 2.

**Table 2:** Palliative Care Team at Moi Teaching and Research Hospital

Full-time employees
Social worker/administrator
Nurse
Clinical officer
Medical officer
Part-time/shared employees
Nutritionist
Physical therapy/occupational therapy
Chaplain
Nurse
Social worker
Data manager

**Figure 1:**
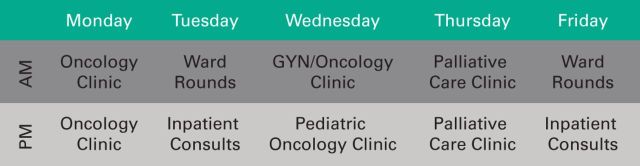
Palliative care clinics and inpatient services. Outpatients are evaluated in a variety of clinics, whereas ward rounds focus on existing inpatients. Inpatient consult time generally focuses on evaluation of new referrals. GYN, gynecology.

The delivery of palliative care for patients with cancer at the MTRH is currently limited to hospital- and clinic-based consultation. The schedule is depicted in Figure 1 with time dedicated to inpatient and outpatient services. Palliative care was intentionally located adjacent to the oncology clinics to facilitate joint evaluations. A stand-alone palliative care clinic is also held once per week.

MTRH personnel and AMPATH-Oncology palliative care physicians have implemented an assessment tool and tracking system to better understand the needs of their patients. The form seeks to identify the physical, spiritual, financial, and social challenges of patients with cancer and their families. The four-page assessment form is summarized in Table 1. During the encounter, the patient and family meet with members of the interdisciplinary palliative care team that includes a medical or clinical officer, social worker, and nurses.

**Figure 2:**
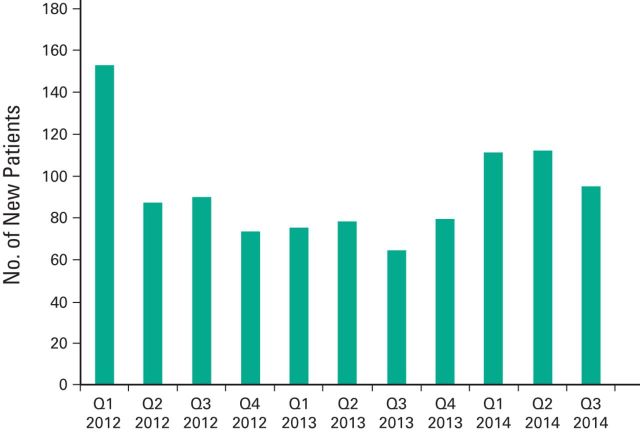
Number of new consults per month. Referrals to the palliative care team are generally equally split between inpatient and outpatient referrals. Q1, first quarter.

**Figure 3:**
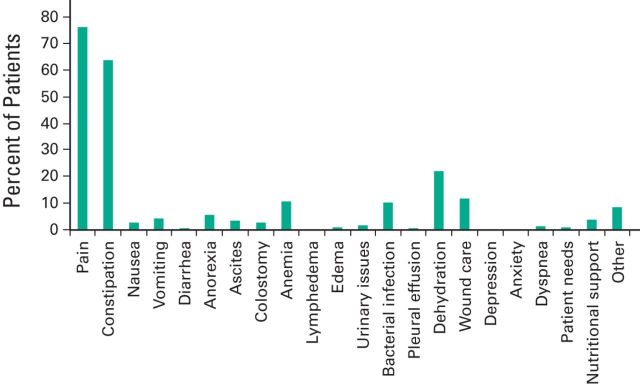
Symptoms assessed during initial evaluation and follow-up visits. The graph shows the percentage of patients with symptoms at the time of evaluation. The data were recorded during 1,909 visits; patients often express more than one symptom at the time of evaluation. Symptoms expressed by the patient that were not listed on the intake sheet were recorded as “Other.”

Formal tracking of the palliative care team's activities began in January 2012. As shown in Figure 2, there has been a steady influx of referrals, with new patient assessments averaging 35 per month in 2014. Between January 2012 and September 2014, 1,017 new patients were evaluated and 1,244 patients were seen in follow-up. The clinical characteristics of the patients are listed in Table 3. The predominant reason for referral is symptom management; 75% of patients were referred for treatment of pain (Fig 3). Constipation was identified by more than 60% of patients. Interestingly, nausea and vomiting were present in only 2.3% and 4.4% of patients, respectively. Meanwhile, dehydration was a common complaint noted in 22.1% of patients. Although patients were asked about anxiety and depression, these were not major symptoms that patients noted during their evaluation. Concerned that the existing questionnaire does not adequately assess psychological stressors, the team will implement the African Palliative Care Association African Palliative Outcome Scale in May 2015.^12^ The use of this validated tool will also foster efficacy assessments and potential research studies as the program continues to grow.

**Table 3:** Characteristics of Palliative Care Patients

Characteristic	No.	%
Sex (all visits)		
Male	899	46.1
Female	1,050	53.9
Age at first evaluation (years)		
0-10	72	7
11-20	84	8
21-30	81	8
31-40	180	17
41-50	189	18
51-60	204	19
61-75	172	16
≥ 76	90	8
Diagnosis		
Cervical cancer	118	16
Esophageal cancer	76	10
Breast cancer	74	10
Hepatocellular carcinoma	45	6
Kaposi sarcoma	35	5
Adenocarcinoma*	34	5
Acute myeloid leukemia	34	5
Acute lymphocytic leukemia	33	4
Stomach cancer	29	4
Pancreatic cancer	25	3
Prostate cancer	24	3
Ovarian cancer	22	3
Squamous cell carcinoma†	20	3
Non-Hodgkin lymphoma	18	2
Rectal cancer	17	2
Osteogenic sarcoma	17	2
Colon cancer	16	2

* Data collected between January 1, 2012, and September 30, 2014.

† Primary site not specified.

### Education and Training

A study of the prevalence and clinical correlates of pain conducted at the MTRH from March to July 2011 noted that 66% of inpatients had undertreated pain, with the highest pain scores noted in older adults as well as patients with HIV and cancer.^13^ The palliative care team initiated a series of educational programs to increase the clinical competency of MTRH personnel regarding the principles of palliative care. The first training was offered in 2011 with 29 individuals participating in a 5-day training session. Training manuals were provided by the Kenya Hospices and Palliative Care Association (KEHPCA). The second training occurred in 2012, with 32 individuals attending. In March 2013, the MTRH palliative care team participated in palliative care training with the United Kingdom-based Tropical Health Education Trust (THET),^14^ a program implemented by the University of Edinburgh, Makerere University, and the African Palliative Care Association in close collaboration with KEHPCA. THET identified three hospitals in Kenya (MTRH, Nyeri, and Homa Bay) for training, and approximately 100 individuals were trained at MTRH. The MTRH palliative care team also contributes to the health sciences curriculum at Moi University.

Continuing education of the palliative care team is also an ongoing priority. The team actively participates in KEHPCA and consistently attends their annual meeting. The team has presented three to four abstracts per year since 2012. Palliative care physicians from the United States spend approximately 3 to 4 weeks per year in Eldoret making rounds with the team. THET palliative care clinical workers also spent the first 2 months of 2014 as part of the MTRH team. In addition, THET provided funds for tuition to enable the team nurse to enroll in an 18-month palliative care diploma program at Nairobi Hospice.

### Barriers to Cancer Pain Management

Limited availability of opioids in Kenya and throughout much of the African continent was the norm^15–17^at the inception of the AMPATH-Oncology palliative care program. A 2010 report from the Human Rights Watch titled “Needless Pain: Government Failure to Provide Palliative Care for Children in Kenya” reported that morphine was available in only seven of the 250 Kenyan public hospitals. The available morphine was sufficient to treat only 1,500 patients; for context, the Treat the Pain initiative estimated that 51,262 Kenyans with HIV and cancer died in moderate to severe pain around this time.^18,19^ In Kenya, KEHPCA has been a key organization in the effort to improve opioid availability. They have partnered with 23 freestanding hospices and 21 palliative care programs to provide education to medical personnel on the proper use of morphine. In addition, they have worked closely with the Kenyan Ministry of Health to purchase morphine for distribution to hospices and palliative care programs that now reach an estimated 30,000 patients with moderate to severe pain.^20^

At this writing, the cost of morphine is significantly subsidized; a week's supply costs 420 Kenyan shillings (approximately US$5.25) for a dose of 10 mg of liquid morphine four times per day. The palliative care social workers can waive the cost if patients have insufficient funds. Morphine is not available in most clinics or public pharmacies, which leaves the palliative care team, in collaboration with the AMPATH-Oncology pharmacy, as a major dispenser of morphine. Pain control is a challenge for some patients who must travel 2 to 3 hours for medication refills. Moreover, pain control is hindered by the limited choice of opioids. Long-acting morphine is not readily available. Transdermal fentanyl is prohibitively expensive for the vast majority of Kenyans, significantly complicating pain management for those with renal failure or other contraindications to morphine.

Palliative radiation therapy is a pressing need in Eldoret. At this time, patients must travel to Nairobi or outside the country, which makes the cost prohibitive for most patients. The AMPATH-Oncology program is currently building a radiation oncology facility in Eldoret that will improve access. Because the cost for radiation therapy has not been established, we do not yet know whether the cost of treatment will be within the means of most Kenyans.

### Socioeconomic Barriers

The World Bank estimated that 45.9% of Kenyans were below the poverty line in 2005.^21^ There appears to be some improvement, with the 2012 estimate at 43.4%.^22^ Nevertheless, the poverty rate is predicted to be above 30% through 2030. This suggests that comprehensive cancer care may remain beyond the reach of most individuals. This includes individuals covered by the National Hospital Insurance Fund (NHIF), the governmental insurance plan. The cost is 160 to 320 Kenyan shillings per month (approximately US$2.00 to US$4.00), which will cover hospital admissions and basic laboratory and medical treatments but excludes chemotherapy and radiation therapy. As a result, many families with insurance decline treatment or interrupt therapy prematurely because of financial constraints. When this occurs, care is frequently shifted to the palliative care team.

The financial structure of health care in Kenya presents several unique challenges. Evaluations or procedures typically performed as outpatient services in most HICs are performed as inpatient services, which often require long hospital stays and incur significant expenses. This complicates hospital release, because public hospitals will not discharge patients until the hospital bill is paid in full. This requirement also applies to individuals who die while in the hospital; release from the morgue requires that outstanding bills be paid. A hospital committee can waive fees but the process is complex and further adds to the hospital stay. The palliative care team expends a lot of effort (in both time and emotion) helping disadvantaged patients navigate this complex system to honor their wish to spend their last days at home or in hospice.

Psychosocial support is further complicated by the complexity of Kenyan families in which men often have more than one wife. The NHIF will cover only one wife, so palliative care social workers are challenged to determine which wife was listed during insurance enrollment. Furthermore, only those children of the enrolled wife are insured. The financial realities lead to children with curable cancers presenting late or relapsing because of noncompliance with treatment regimens.

## DISCUSSION

Delivering palliative care in LMICs such as Kenya presents a combination of shared and unique challenges compared with those encountered in HICs. Communicating prognostic information to patients and their families is a universal challenge for health care professionals. The impact of cancer on the financial stability of families is also a shared challenge, although the majority of patients in HICs struggle financially after treatment whereas many in LMICs do not receive treatment because of their financial situation.

The palliative care team described here is hospital based and focused on the care of patients with cancer. To improve services, an inpatient palliative care unit would facilitate the management of complex symptoms, but an assessment of the cost (or cost savings) will be required to determine whether dedication of already limited resources is warranted.

An inpatient hospice unit is not offered at MTRH, but the team has used other hospice facilities when possible. The team has used Kimbilio Hospice, a faith-based nongovernmental agency. Unfortunately, this facility is in a location far from many in the MTRH catchment area; there is a significant need for additional inpatient hospices in Kenya.

Outpatient care is generally not covered by the NHIF insurance, so revenue for MTRH to support home hospice services is not currently available. The team has tried to meet these limitations by manning a 24-hour hotline to help patients at home manage their symptoms. The team also has an innovative program held once a month in which patients and their families are invited to spend a morning discussing pain management, nutrition, and insurance, and a chaplain is present to discuss issues of faith.

The increasing availability of intensive care units (ICUs) in Kenya challenges physicians to develop selection criteria to prioritize patients for ventilator support. Although the logistic and ethical challenges for selecting patients for life support in Kenya are part of ongoing discussions,^23^ palliative care teams are now beginning to face the questions around withdrawal of life support. ICU beds at MTRH are a relatively new and limited resource. Experience in prognostication combined with limited legal precedence challenges both the ICU team and palliative care physicians in caring for individuals maintained on life support who are not expected to recover.

In summary, palliative care services in Eldoret, Kenya, have become a key component of its comprehensive cancer treatment program. A 24-hour hotline facilitates symptom management for outpatients and identifies patients in need of transfer to an inpatient hospice. Other opportunities for improving care are patient education and implementation of tools that can provide patients with guidance on self-management of pain.^24^ Furthermore, expanded outpatient and inpatient hospice services would improve end-of-life care for many patients. Ultimately, the economic health of Kenya will be key to improving the outlook for Kenyans with cancer. The success of current cancer prevention efforts, earlier diagnosis, and treatment of cancer, along with coverage of chemotherapy by the national insurance fund will provide access to cancer treatments, thus allowing palliative care providers the opportunity to focus on quality of life during effective treatment rather than the current focus on end-of-life care.
